# Design Paper: A Prospective, Multicenter, Single-arm, Phase II Trial of Tailored Axillary Surgery in Patients with Clinically Node-positive Breast Cancer in the Upfront Surgery Setting

**DOI:** 10.31662/jmaj.2024-0187

**Published:** 2024-12-20

**Authors:** Yasuaki Sagara, Kaori Terata, Takehiko Sakai, Shin Takayama, Dai Kitagawa, Tsuguo Iwatani, Takahiro Tsukioki, Mami Ogita, Naoko Sanuki, Masayuki Yoshida, Hitoshi Tsuda, Seiichiro Yamamoto, Hiroji Iwata, Tadahiko Shien

**Affiliations:** 1 Department of Breast Surgical Oncology, Social Medical Corporation Hakuaikai Sagara Hospital, Kagoshima, Japan; 2 Department of Breast and Endocrine Surgery, Akita University Hospital, Akita, Japan; 3 Department of Surgical Oncology, Breast Oncology Center, Cancer Institute Hospital of JFCR, Tokyo, Japan; 4 Department of Breast Surgery, National Cancer Center Hospital, Tokyo, Japan; 5 Department of Breast Surgical Oncology, National Center for Global Health and Medicine, Tokyo, Japan; 6 Department of Breast and Endocrine Surgery, Okayama University Hospital, Okayama, Japan; 7 Department of Radiology, The University of Tokyo Hospital, Tokyo, Japan; 8 Department of Radiology, Yokkaichi Municipal Hospital, Yokkaichi, Japan; 9 Department of Diagnostic Pathology, National Cancer Center Hospital, Tokyo, Japan; 10 Department of Basic Pathology, National Defense Medical College, Japan; 11 Shizuoka Graduate University of Public Health, Shizuoka, Japan; 12 Department of Advanced Clinical Research and Development, Nagoya City University, Nagoya, Japan

**Keywords:** tailored axillary surgery, breast cancer, lymph node dissection, surgical complications, quality of life

## Abstract

**Introduction:**

This prospective, multicenter, single-arm Phase II trial investigates the feasibility and the safety of tailored axillary surgery (TAS) in patients with clinically node-positive breast cancer who are undergoing upfront surgery. The trial aims to establish the criteria for safely omitting axillary lymph node dissection (ALND) in these cases, potentially shifting breast cancer management by minimizing surgical complications and preserving the patients’ quality of life (QOL).

**Methods:**

The study includes patients who were diagnosed with invasive breast cancer, particularly those with limited metastatic lymph nodes. The primary objective of this work is to determine the specific combination of clinical and pathological factors that would result in a non-TAS lymph node metastasis proportion of less than 10%. The secondary objectives include assessing the identification rate of the metastatic lymph nodes, the incidence of upper limb lymphedema, and the QOL measures.

**Results:**

The results will identify the patient eligibility criteria for the Phase III TAS trial, potentially allowing the omission of ALND in selected patients. This may lead to reduced surgical complications and better preservation of the QOL of patients with breast cancer.

**Conclusions:**

The trial’s outcome will contribute to the development of the criteria for safely omitting ALND in certain patients with clinically node-positive breast cancer. This approach aims to enhance breast cancer management by reducing surgical burden and improving the patient outcomes.

jRCTs: 061220113

## Trial Background and Rationale

In 2019, the Japanese Ministry of Health reported that the number of women with breast cancer reached 97,142, of which 14,839 breast cancer cases led to deaths ^(1)^. Despite a global increase in the breast cancer incidence, breast cancer prognosis has been improved through early detection via screening and comprehensive treatment approach, including systemic therapy, surgery, and radiotherapy ^(2), (3)^.

The primary objective of breast cancer surgery is to achieve local control, avert metastasis, and evaluate the breast cancer stage. Standard procedures include excising the breast tumor with adequate margins and conducting sentinel lymph node biopsy (SLNB) and/or axillary lymph node dissection (ALND). ALND has been the uniform approach because of the challenges in exclusively identifying metastatic lymph nodes. However, ALND leads to complications, such as lymphedema, reduced arm mobility, numbness, and pain. SLNB, which entails removing only the initial lymph nodes affected by the breast cancer metastasis, has become the standard less-invasive method for staging breast cancer in cases without a clinically apparent lymph node metastasis (cN0) ^(4), (5), (6)^. In 2020, SLNB was performed in 73.6% of women who were newly diagnosed with breast cancer from the Japanese National Clinical Database ^(7)^.

The ACOSOG Z0011 trial assessed the necessity of ALND in patients with cN0 who were found to have lymph node metastasis by SLNB ^(4)^. It demonstrated that SLNB alone was equally effective as ALND in terms of local recurrence and overall survival while reducing the surgical complications associated with ALND. The trial results influenced breast cancer treatment guidelines, suggesting that for patients with fewer than three lymph node metastases, avoiding ALND may be the preferred approach.

The current clinical challenge is establishing a standard axillary procedure for clinical lymph node metastasis (cN+) cases. ALND has been a standard approach for breast cancer patients with cN+. However, several clinical trials have investigated the feasibility of omitting ALND in patients initially diagnosed with clinically node-positive (cN+) breast cancer. In this context, the emerging technique of targeted axillary dissection (TAD) is gaining attention as a safe approach for these cN+ cases that transition to clinically node-negative (cN0), followed by preoperative systemic therapy ^(8), (9), (10)^. TAD involves the removal of the initially swollen lymph nodes marked with a clip in conjunction with the sentinel lymph nodes. By focusing on the regions most susceptible to breast cancer, TAD aims to reduce the false negative rate, potentially bringing it to below 10% ^(11)^.

The breast cancer treatment strategies vary based on the expression of hormone receptors (HR) and HER2 status classified into four distinct subtypes. These treatments include chemotherapy, endocrine therapy, and anti-HER2 therapy tailored to each subtype. For HER2-positive and triple-negative breast cancers, neoadjuvant systemic therapy is the standard approach. This is followed by postoperative systematic therapy according to the response of neoadjuvant therapy ^(12), (13)^. In contrast, HR-positive/HER2-negative breast cancers, which account for approximately 70% of cases, often show a lower response rate to systemic therapy ^(2)^. It leads to a low conversion rate from a clinically node-positive (cN+) to a clinically node-negative (cN0) status ^(14)^.

Consequently, we aim to examine the feasibility and the safety of tailored axillary surgery (TAS) in cases where surgery precedes systemic therapy in cN+ patients. We defined TAS as the removal of labeled and swollen metastatic lymph nodes plus SLNB. However, when TAS was performed for cN+ cases, there were scarce data regarding the identification rates of the marked metastatic lymph nodes and the sentinel lymph nodes and the axillary recurrence rate. Moreover, the clinical factors that would serve as the criteria for the eligibility of TAS, such as understanding, in which patients the lymph nodes not included in TAS (non-TAS lymph nodes) would not have metastases, are also unclear. Thus, this prospective trial aims to validate the TAS technique and explore the feasibility of safely forgoing ALND in specific clinical scenarios. The study protocol was approved by the Central Institutional Review Board of Okayama University before patient accrual was started. The patient accrual began in April 2023.

Objectives: This trial aims to establish a surgical procedure of TAS among patients with cN+ who are undergoing upfront surgery and determine the appropriate criteria for a Phase III TAS trial to safely omit ALND in the context of limited metastatic LNs other than those resected lymph nodes by TAS. The hypothesis of this study is as follows: preoperative tumor size, number of metastatic lymph nodes on preoperative imaging, number of lymph nodes removed by TAS, and other factors that can identify targets with a low percentage of non-TAS lymph node metastases; TAS removes lymph nodes, including a labeled metastatic lymph node (identification rate > 90%).

Our objective is to determine the specific combinations of clinical and pathological factors resulting in a proportion of non-TAS lymph node metastasis being less than 10% in patients undergoing TAS.

## Endpoints

Primary objective: To determine the proportion of non-TAS lymph node metastasis in patients undergoing TAS considering the following clinicopathologic factors for investigating non-TAS lymph node metastasis:

・number of lymph node metastases suspected in preoperative imaging (one, two, and three nodes);

・number of lymph nodes removed via TAS (one, two, three, four, or more nodes);

・number of metastatic lymph nodes removed in TAS;

・tumor size (cT1, cT2, and cT3);

・presence of invasive lobular carcinoma (yes, no); and

・methods used for marking metastatic lymph nodes (wire, dye or India ink, clip + dye or India ink, clip + wire).

Secondary objectives: To determine the proportion of TAS identification (cases where at least one metastatic lymph node is included in the lymph nodes removed by TAS/all eligible cases), identification rate of the marked metastatic lymph node (one node), identification rate of metastatic lymph nodes for each marking method, incidence rate of upper limb lymphedema, quality of life, Quick DASH, and FACT-B + 4 ARM subscale.

## Eligibility Criteria

### Inclusion criteria

The participants must satisfy all of the following conditions:

(i) women aged between 18 and 74 years with histologically confirmed invasive breast cancer;

(ii) node-positive status confirmed through pathology;

(iii) preoperative imaging, including ultrasound, MRI, and PET, indicates three or fewer axillary lymph nodes suspected of metastasis;

(iv) scheduled for primary surgery without preoperative systemic therapy;

(v) the most suspected metastasis lymph node is confirmed as metastatic through cytology or needle biopsy;

(vi) there is no evidence of metastasis in levels II, III, Rotter, internal mammary, and supraclavicular lymph nodes (LNs);

(vii) the clinical classification of the primary tumor is T1-T3;

(viii) an ECOG performance status of 0 or 1;

(ix) no bilateral breast cancer, either synchronous or metachronous; and

(x) the patient could receive standard postoperative radiotherapy and systemic therapy.

### Exclusion criteria

Individuals meeting any of the following conditions will be excluded from the study:

(i) presence of active multiple cancers;

(ii) infectious diseases requiring systemic treatment;

(iii) women who are pregnant might be pregnant within 28 days postpartum or are breastfeeding;

(iv) mental illness or psychiatric symptoms that significantly impair daily life are considered to hinder participation in the trial;

(v) ongoing systemic administration (oral or intravenous) of steroid drugs or other immunosuppressive medications;

(vi) uncontrolled diabetes mellitus, hypertension, unstable angina, or a history of myocardial infarction within the past 6 months;

(vii) conditions, such as interstitial pneumonia, pulmonary fibrosis, severe pulmonary emphysema, or any combination thereof; and

(viii) history of axillary surgery or radiotherapy.

## Study Design

This is a multicenter, prospective, single-arm Phase II clinical trial.

### Intervention summary of the clinical trial protocol

#### Registration and surgery

**Figure 1.**
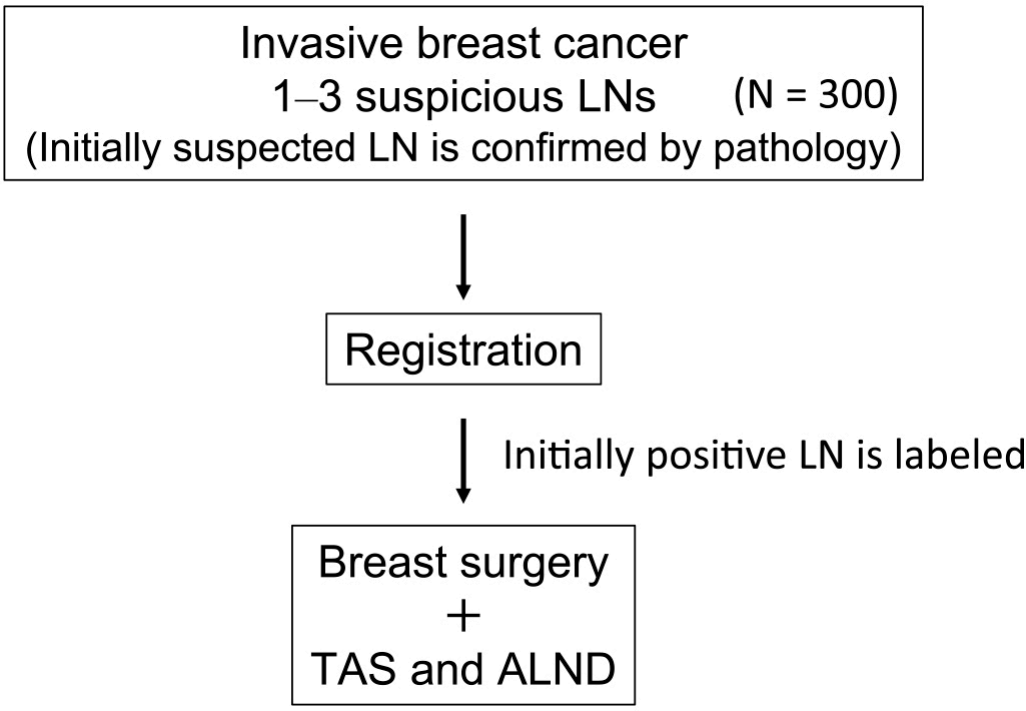
Prospective study design. LN, lymph node; TAS, tailored axillary surgery; and ALND, axillary lymph node dissection.

Informed consent was obtained from all patients prior to their participation in the trial. Before trial registration, the most suspected lymph node underwent cytology or needle biopsy to confirm metastasis (Figure 1). Standard breast surgery (conserving or mastectomy) was conducted, with reconstruction based on the patient’s preference. Marking of the suspicious lymph node (one node) was performed using clips, wires, dyes, or India ink. The surgeon could choose a marking method for optimal identification, supplemented by imaging techniques, if necessary.

**Figure 2.**
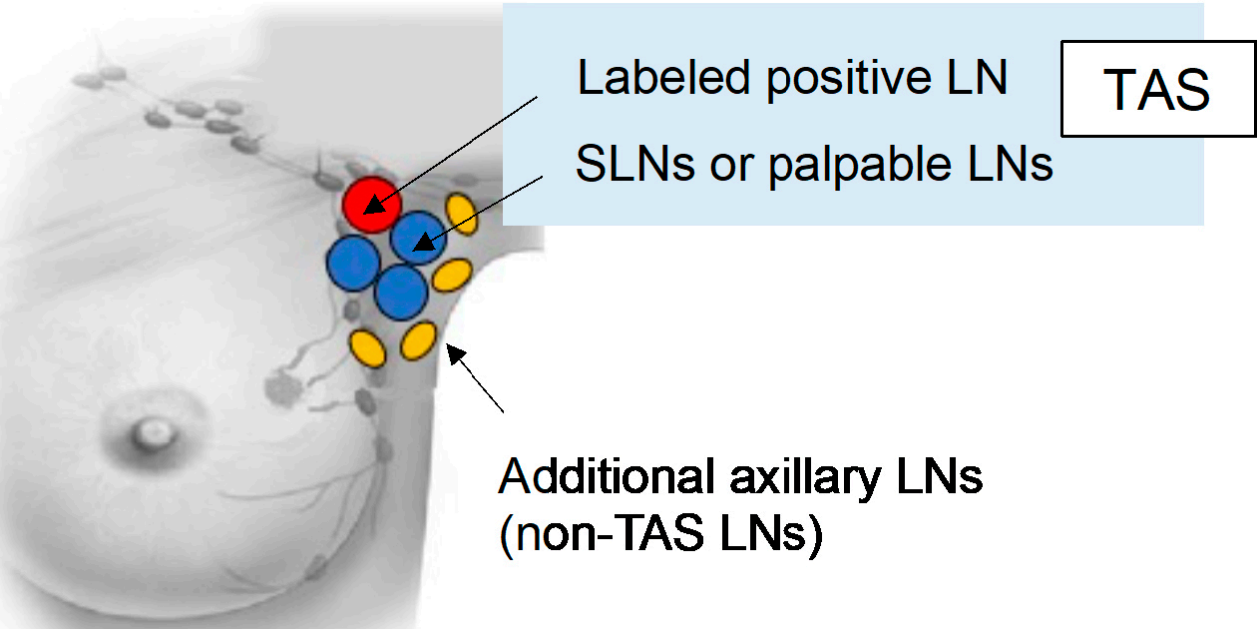
Schema of tailored axillary surgery and additional axillary lymph node dissection in the prospective trial. LN, lymph node; and TAS, tailored axillary surgery.

During the surgical procedure, the surgeon excises the premarked node, the sentinel lymph nodes, and any axillary LNs that are palpably suspicious. The surgeon should carefully avoid removing the normal LNs without a metastasis feature. Subsequent ALND involves levels I and II for identifying non-TAS lymph node metastases (Figure 2).

#### Pathological examination

The labeled positive lymph node, sentinel lymph node(s), and palpably suspicious LNs are sliced into two pieces. Both pieces are then histologically examined. For the subsequent axillary LNs obtained with ALND, one representative cut surface is histologically examined.

#### Radiotherapy

In cases where postoperative chemotherapy is indicated, chemotherapy is generally preceded. After its completion, the patient receives radiotherapy promptly if there are no issues with the his/her overall condition. If postoperative chemotherapy is not planned, the patient receives radiotherapy after the surgical wound has healed. The patient receives radiotherapy in conventionally or moderately hypofractionated sessions, with a tumor bed boost radiation applied per facility protocol. Treatment planning is based on CT scans with three-dimensional conformal radiotherapy or intensity-modulated radiation therapy. The target volume includes the entire breast (after conserving surgery) or the chest wall (after mastectomy) and the regional LNs depending on the number of lymph node metastases.

#### Systemic therapy

According to the cancer subtype and stage, postoperative systemic therapy, including chemotherapy, endocrine therapy, and target therapy, is initiated promptly as per the established clinical guidelines.

## Statistical Methods

This study aims to identify the treatment factor combinations yielding a non-TAS metastasis-positive rate of ≤10% when the surgeon performs TAS. To statistically reject a non-TAS metastasis-positive rate ≥10%, a single-factor combination requires a sample size of 167 cases (with a one-sided type I error rate of 5%, a power of 70%, and an expected value of 5%). Conducting studies for every factor combination is impractical. We employed herein a regression model with the non-TAS metastasis-positive rate as the dependent variable and treatment selection factors as independent variables to enhance the estimation accuracy. This model aids in identifying the factor combinations with a predicted non-TAS metastasis-positive rate ≤ 10%.

A minimum sample size of 10 times the number of predictive factors is recommended for the regression analysis. Therefore, we will apply the model after accumulating data from 60 cases. Factor combinations predicted by the regression model to have a non-TAS metastasis-positive rate with a 90% confidence interval upper limit <10% will be considered potential candidates. If the predictive value’s precision is deemed insufficient, an additional 60 cases will be incorporated iteratively, not exceeding 300 cases.

## Key Milestones:

・Participant recruitment: Recruitment of participants who meet the eligibility criteria has started from the jRCT publication date of May 29, 2023 and will close by February 28, 2027. The study is projected to conclude in 2034.

・Treatment administration and data collection: Participants receive surgery for breast cancer, and researchers collect the data on the patient’s background, clinical information, and study endpoints. These data are collected using REDCap^®^ at the Center for Innovative Clinical Medicine at Okayama University.

・Monitoring and safety oversight: The principal investigator annually reports study implementation status and safety assessments to the administrator and certified clinical research review committee at Okayama University. In case of serious adverse events, responsible physicians must inform participants, take necessary actions, and promptly report to both the administrator and the principal investigator, who then report to the review committee and other medical institutions.

・Initial data analysis: A preliminary statistical analysis using a regression model is performed after accruing 60 cases. The details of the statistical method are described above.

・Decision-making for further development: Based on this trial’s result, we identify the patients’ eligibility criteria and decide whether or not to proceed to Phase III. We hypothesize that a non-TAS lymph node metastasis rate of less than 10% would be a threshold for considering to proceed to Phase III. Patients with certain tumor sizes, limited numbers of metastatic LNs as identified by preoperative imaging, and favorable histological subtypes may be identified as ideal candidates. We plan to perform a detailed subgroup analysis poststudy to identify the population with the most benefit and the lowest risk, which will help us narrow down the target population for Phase III.

・Regulatory discussions: If we decide to proceed, discussions with regulatory authorities, including the methods of TAS, may occur to plan for Phase III.

## Discussion

The ongoing Phase II prospective trial plays a crucial role in the evolution of breast cancer treatment for patients with positive axillary LNs. Its primary objective is to establish the clinical criteria for selecting breast cancer patients who may safely forgo ALND and qualify for a Phase III clinical trial focused on TAS. It is a significant step in the field because the outcomes from this trial will directly determine the optimal eligibility criteria for the subsequent Phase III trial. This latter trial explores the possibility of completely excluding ALND for certain patients, a notable shift from current practices.

The potential effect of this study is substantial. If successful, the trial could lead to a paradigm shift in how breast cancer is managed, especially in patients with positive LNs. The TAS implementation in these patients could enable a significant number of cases to avoid the surgical complications associated with ALND, such as upper limb lymphedema, deterioration in the QOL, and reduced arm mobility. By comparing these outcomes in patients undergoing ALND in the Phase II trial with those undergoing TAS without ALND in the Phase III trial, the study aims to provide additional evidence for the TAS benefits.

In the context of the current research, the TAXIS trial (NCT03513614) is an essential referential study ^(15)^. This trial targets Stage II/III cases with/without neoadjuvant systemic therapy, evaluates the safety and the utility of TAS for cN+ cases, and involves randomizing subjects into TAS and TAS + ALND groups. The accrual completion of the TAXIS trial is planned for 2025, with the primary endpoint analysis expected in 2029. It will provide valuable data on disease-free survival as the primary endpoint. However, the TAXIS trial has a different focus from the ongoing Phase II trial because it includes a broader range of patients. Moreover, the TAXIS trial’s feasibility study has shown that many patients in the TAS + ALND group have non-TAS lymph node metastases ^(16)^. It raises questions about whether local control could be achieved in patients undergoing only TAS, as there may be numerous residual lymph node metastases in the axilla.

In contrast, the ongoing Phase II trial aims to refine the patient selection criteria for TAS, focusing on those who could safely receive TAS while minimizing the risk of excessive residual metastatic nodes. This tailored approach could offer patients more precise and less invasive treatment options, consequently setting the stage for the Phase III trial. A comparison of these trials will provide valuable insights into the effectiveness and the safety of TAS in different patient populations, potentially influencing the future breast cancer treatment protocols and improving the patient outcomes.

In conclusion, the ongoing Phase II prospective trial represents a pivotal step in establishing the ideal clinical criteria for selecting patients who can safely opt for TAS without ALND. By potentially reducing the need for ALND, this trial offers a promise of not only minimizing surgical complications, but also preserving the QOL for breast cancer patients.

## Article Information

### Conflicts of Interest

None

### Sources of Funding

This work was supported by a research grant from the [Okayama University Hospital].

### Author Contributions

Work concept or design: Yasuaki Sagara, Kaori Terata, Takehiko Sakai, Shin Takayama, Dai Kitagawa, Tsuguo Iwatani, Takahiro Tsukioki, Mami Ogita, Naoko Sanuki, Masayuki Yoshida, Hitoshi Tsuda, Hiroji Iwata, Tadahiko Shien

Analysis: Seiichiro Yamamoto

Final version approval, work draft, or critical review for important intellectual content; agreement to be held accountable for all aspects of the work, and ensuring that questions related to the accuracy or integrity of any part of the work are appropriately investigated and resolved: All authors

### Approval by Institutional Review Board (IRB)

CRB22-007 from the Okayama University Clinical Research Review Committee

### Registration of the Protocol

The protocol was registered on the Japan Registry of Clinical Trials (JRCT) website (protocol ID jRCTs061220113) on May 29, 2023. The details are available at the following web address: https://jrct.niph.go.jp

### Participating Institutions

This study will be conducted at following 41 institutions mainly belonging to the Japan Clinical Oncology Group-Breast Cancer Group; National Hospital Organization Hokkaido Cancer Center, Akita University Hospital, Tohoku University Hospital, Fukushima Medical University, University of Tsukuba, Jichi Medical University, Gunma Prefectural Cancer Center, Saitama Cancer Center, National Cancer Center Hospital East, Chiba Cancer Center, National Cancer Center Hospital, Tokyo Metropolitan Cancer and Infectious Diseases Center Komagome Hospital, National Hospital Organization Tokyo Medical Center, National Center for Global Health and Medicine, Cancer Institute Hospital of JFCR, Toranomon Hospital, Saitama Medical Center, Nagoya City University Hospital, Seirei Sakura Citizen Hospital, Kanagawa Cancer Center, Kitasato University School of Medicine, Shizuoka General Hospital, Shizuoka Cancer Center, Aichi Cancer Center Hospital, Nagoya Medical Center, Hamamatsu University School of Medicine, Nagoya University Hospital, National Hospital Organization Osaka National Hospital, Osaka International Cancer Institute, Kansai Medical University Hospital, Yao Municipal Hospital, Kure Medical Center Chugoku Cancer Center, Mie University Hospital, Okayama University Hospital, Hiroshima University Hospital, National Hospital Organization Fukuyama Medical Center, Yamaguchi University Hospital, Shikoku Cancer Center, Kitakyushu Municipal Medical Center, National Kyushu Cancer Center, and Hakuaikai Sagara Hospital.
